# Hematological indices and their correlation with glucose control parameters in a prediabetic rat model

**DOI:** 10.14202/vetworld.2022.672-678

**Published:** 2022-03-24

**Authors:** Desak Gede Budi Krisnamurti, Erni H. Purwaningsih, Tri Juli Edi Tarigan, Vivian Soetikno, Melva Louisa

**Affiliations:** 1 Doctoral Program in Biomedical Sciences, Faculty of Medicine, Universitas Indonesia, Jakarta, Indonesia; 2 Department of Medical Pharmacy, Faculty of Medicine, Universitas Indonesia, Jakarta, Indonesia; 3 Department of Internal Medicine, Faculty of Medicine, Universitas Indonesia, Jakarta, Indonesia; 4 Department of Pharmacology and Therapeutics, Faculty of Medicine, Universitas Indonesia, Jakarta, Indonesia

**Keywords:** diabetes mellitus, high-fat and high-glucose diet, hyperglycemia, neutrophil-lymphocyte ratio, streptozotocin

## Abstract

**Background and Aim:**

Chronic hyperglycemia in prediabetic individuals would progress to diabetes and lead to several systemic disruptions, including hematological parameters. This study aimed to investigate the correlation between prediabetes and hematological indices in a prediabetic rat model.

**Materials and Methods:**

Eighteen male rats were randomly divided into two groups of nine. Prediabetes was induced in nine rats by a 3-week high-fat and high-glucose diet, followed by low-dose streptozotocin (STZ) injection (30 mg/kg body weight). The oral glucose tolerance test was performed, and the fasting blood glucose (FBG) and insulin levels were measured 72 h after STZ administration. The control group of nine rats was given standard diets. At the end of the 3^rd^ week, the animals fasted overnight before blood collection. Blood samples were drawn and used for the analysis of the FBG and fasting insulin levels and glycated albumin to define prediabetes criteria before hematology analysis.

**Results:**

We found a significant increase in the FBG and insulin levels in the prediabetic versus the control group. There were decreases in red blood cells, hemoglobin, and hematocrit levels and red cell distribution in prediabetic rats versus the control. At the same time, a significant increase in the platelet count was observed in the prediabetic group. There was a positive correlation between FBG and lymphocytes and neutrophil-lymphocyte ratio in prediabetic rats. On the other hand, we found a negative correlation between white blood cell count and glycated albumin.

**Conclusion:**

Correlations were found in several hematological parameters in the prediabetic rat models. The changes in hematological indices in prediabetic rats may be further used as a valuable indicator of glycemic control.

## Introduction

Prediabetes is a condition in which the plasma glucose level is above the normal limit yet still below the defined threshold of diabetes mellitus. Individuals with prediabetes have a high risk of developing diabetes. The American Diabetes Association categorizes prediabetic as a spectrum of hyperglycemic conditions based on hemoglobin A1c (HbA1c) or fasting 2-h post load glucose [1]. Prediabetic individuals are at higher risk of developing diabetes mellitus [2]. In a 3-5-year duration, approximately one-fourth of individuals with prediabetes will develop diabetes at a certain point in time. Moreover, subjects with diabetes will have a higher risk of several systemic disturbances and cardiovascular diseases [3]. Chronic hyperglycemia disrupts various blood cell components, resulting in several microvascular issues [4]. Diabetes-related circulatory problems are the primary reasons for morbidity and death. The most common morbidity had been associated with coronary heart disease, endothelial dysfunction, and hematological index impairment [5]. The previous study had shown changes in routine hematological counts concerning diabetes mellitus. At the same time, disruptions in hematological parameters are followed by increased inflammatory marker levels, susceptibility to clot formation, and microcirculatory abnormalities [6]. Following the circulatory disturbance, advanced glycation end products were activated, generating oxidative stress systemically, altering membrane and vasculature structures and hemodynamic characteristics [7].

Controlling hyperglycemia is the main suggestion for the prevention of diabetes complications. Hyperglycemia and insulin resistance cause an increase in platelet reactivity through direct effects and by promoting glycation of platelet proteins on the platelet surface [8]. This glycation decreases membrane fluidity and tends to activate the platelets. Platelets that have been stimulated produce a variety of specific mediators that promote engagements between blood cells and the endothelium, by directly regulating the activation of the endothelium and white blood cells (WBCs). In agreement with these findings, prior research suggested that positive glycemic management reduces platelet activation due to the modulation of protein kinase C activation. The mean platelet volume (MPV) is the average size of platelets, showing platelet function and activation, which may play a role in developing vascular complications in diabetes [9]. A previous study [10] has found a significant association between MPV and fasting blood glucose (FBG) in individuals with diabetes. The MPV levels were shown to be significantly higher in individuals with diabetes than in nondiabetic individuals, which suggested that these are linked to the levels of postprandial plasma glucose and HbA1c [10]. Thus, hematological indices may play a role in the early development of Type 2 diabetes mellitus (T2DM) from the prediabetic condition. A prior study proposed that the early correction of hyperglycemia may prevent the pathogenic processes associated with increased oxidative stress. A previous study suggested that earlier normalization of hyperglycemia inhibits the pathological processes related to increased oxidative stress generation and intracellular protein damages [11].

Although studies have signified the hematological disturbances in insulin resistance and diabetes mellitus, whether the process started from prediabetes is not well understood [12]. To date, a small number of studies indicate that there may be a change in routine hematological counts in hyperglycemic conditions [6]. Hematological indices are valuable indicators for use in monitoring and managing diabetic individuals.

Hence, the present study aimed to examine the correlation between hematological and glucose control parameters in prediabetic rats induced by a high-fat and high-glucose diet (HFD-G) followed by streptozotocin (STZ).

## Materials and Methods

### Ethical approval

The Ethics Committee approved the study protocol from the Faculty of Medicine Universitas Indonesia with No. KET-701/UN2.F1/ETIK/PPM. 00.02/2020.

### Study period and location

The study was conducted from January 2021 to June 2021 in Pharmacokinetics Laboratory and Integrated Laboratory, Faculty of Medicine, Universitas Indonesia.

### Animals

We used 18 male rats of Wistar strain aged 4 weeks, weighing 80-100 g. The rats were obtained from the National Institute of Health Research and Development, Indonesian Ministry of Health. In this study, we used male rats only to exclude the possibility of hormonal factors in female rats that may influence the results. They were kept in standard rat cages with controlled temperature (22±3°C), 50-70% relative humidity, and under 12 h of light-dark cycles. Study rats were kept acclimatized for 1 week before the administration of a high-fat diet (HFD) or the control diet.

### Protocols

Rats were randomly divided into two groups of nine. The first group was fed an HFD in combination with a high-glucose level (20% glucose) in drinking water over a 3-week duration, followed by a low dosage of STZ (30 mg/kg body weight [BW] intravenously) to obtain a prediabetic model. The HFD (TestDiet, 58V8 rat chow, Richmond, USA) contained 35.8 wt% carbohydrates, 46.1 wt% fat, and 18.1 wt% protein. The calculated calories obtained from the HFD were 4.60 kcal/g. Streptozotocin (STZ; Sigma-Aldrich, St. Louis, MO, USA) was prepared by dissolving the drug in a 0.01 M citrate buffer with pH 4.5. If the blood glucose level had not reached the prediabetic target, STZ injection would be repeated using 15 mg/kg BW. If, after the second dose, the blood glucose level still did not satisfy prediabetic targets, the rats would not be included in the final analysis.

In the normal control group, rats were fed a standard diet (TestDiet, 5012 rat diet) containing 59.88 wt% carbohydrates, 13.10 wt% fat, and 27.02 wt% protein, followed by saline injection. The calculated calories obtained from the standard diet were 3.07 kcal/g.

After 72 h of STZ injection, an oral glucose tolerance test (OGTT) was performed, and the FBG and insulin levels were measured. The rats were given glucose (2 g/kg BW) orally, and then the glucose level was measured 120 min after glucose administration. Rats with FBG levels of ≥110 mg/dL, fasting insulin levels of ≥25 mlU/L, and 2-h post-load glucose levels (OGTT) of 140-199 mg/dL were classified as prediabetic.

### Blood collection

Three days after the rats met the prediabetic criteria, blood sample (1 mL) was collected from retro-orbital sinus of each rat using an injectable anesthetic agent (ketamine-xylazine) and capillary tube. A capillary was inserted into the medial canthus of the rats’ eyes. Blood from each rat was transferred into a clean ethylenediaminetetraacetic acid (EDTA) tube and plain tube (K3 EDTA Vaculab OneMed, Indonesia). Whole blood from the EDTA tube was then sent to the laboratory for hematological analysis. Blood from the plain tubes was centrifuged at 1,000 x g for 10 min, and then supernatant (serum) was transferred into a clean Eppendorf tube and stored at −80°C until analysis.

### Biochemical assay

The FBG level was measured by the enzymatic colorimetric analysis using a Randox kit and read using an ultraviolet-visible spectroscopy spectrophotometer (Perkin – Elmer Lambda 25, USA). After 120 min of glucose administration (OGTT), the blood glucose levels were estimated using a glucometer (Accu-Check, Roche Diagnostics, USA). The fasting insulin and glycated albumin levels were measured using a rat insulin enzyme-linked immunosorbent assay kit (Thermo Fisher Scientific, USA).

### Hematological assay

The hematological indices, such as Hb, hematocrit (Hct), mean corpuscular volume, mean corpuscular Hb, mean corpuscular Hb concentration, MPV, platelet, red blood cell (RBC), WBC, lymphocytes, and neutrophils, were determined using an automated hematology analyzer (Sysmex KX-21, Japan) in the Integrated Laboratory, Faculty of Medicine, Universitas Indonesia according to the manufacturer’s protocol.

### Statistical analysis

Data were analyzed in GraphPad Prism 9.1.2 (GraphPad Software, USA). The mean and standard error of the mean or standard deviation displayed normally distributed data. The data comparison between the two groups was analyzed using an independent sample *t*-test or the Mann–Whitney *U* test based on the data distribution for nonnormally distributed data. The possible correlation between the glucose control parameters and hematological variables was analyzed using Pearson’s and Spearman’s correlation coefficient. A statistically significant difference was defined at p*<*0.05.

## Results

### Glucose control variables in prediabetic rats versus control

After the 3-week administration of an HFD-G diet followed by a low dosage of STZ injection, the prediabetic variables were determined by measuring the FBG and fasting insulin levels. Subsequently, an OGTT was performed. All data were calculated to compare normal and prediabetic rats, and all data are presented in Table-1. The results showed that the mean FBG level was significantly higher (p=0.0207) in prediabetic rats than in control rats. After performing the OGTT and determining the fasting insulin levels, the glucose levels were higher (p=0.0085) in prediabetic rats than in normal rats.

**Table-1:** Biochemical analysis of prediabetic rats versus control.

Variable	Mean±SEM (Normal)	Mean±SEM (Prediabetic)	p-value
Fasting blood glucose (mg/dL)	139.4±24.09	296.5±35.05	0.0207*
Blood glucose levels 120 min post OGTT (mg/dL)	125.3±2.97	221.6±18.92	0.0085**
Fasting insulin level (μIU/mL)	12.38±2.62	21.29±1.43	0.0055**
Glycated albumin (%)	52.75±1.10	58.93±2.14	0.1200

All data are expressed in meanSEM.

* p<0.05 versus control.

** p<0.001 versus control.

SEM=Standard error of the mean, OGTT=Oral glucose tolerance test

### Comparison of the hematological profile between normal and prediabetic rats

The results of the hematological indices are shown in Table-2. The prediabetic condition decreased the RBC count, Hb and Hct levels, and red cell distribution width (RDW) compared with those in the control group. A significant increase in the platelet count (p=0.0001) was observed in the prediabetic group.

**Table-2:** Hematological parameters in control and prediabetic rats.

Variables	Mean±SD (Control)	Mean±SD (Prediabetic)	p-value
WBC indices			
WBCs (10^3^/μL)	12.13±3.25	12.42±2.87	0.845
Lymphocytes	60.56±9.17	59.00±7.83	0.7037
Neutrophils	25.22±12.37	34.22±12.48	0.1439
NLR	0.432±0.25	0.61±0.29	0.1799
RBCs indices			
RBCs	10.16±1.45	8.61±0.60	0.0090*
Hemoglobin (g/dL)	17.11±2.07	14.47±0.82	0.0026**
Hematocrit (%)	54.11±5.26	45.78±2.78	0.0007**
MCV (fL)	53.67±2.92	53.11±1.27	0.6074
MCH (pg)	17±0.71	16.78±0.67	0.6822
MCHC (g/dL)	31.44±0.88	31.89±0.33	0.1244
RDW (fL)	22.36±4.55	18.59±0.96	0.0273*
Platelet indices			
Platelet	839.7±98.01	1341±204.8	0.0001**

Data are shown in Mean±SD.

* p<0.05 versus control

** p<0.005 versus control

***p<0.001 versus control versus control. SD=Standard deviation, WBC=White blood cell, RBCs=Red blood cells, NLR=Neutrophil to lymphocyte ratio, MCV=Mean corpuscular volume, MCH=Mean corpuscular hemoglobin, MCHC=Mean corpuscular hemoglobin concentration, RDW=Red cell distribution width

### Correlations of the hematological indices with the FBG, insulin, and glycated albumin levels

The correlation of insulin with neutrophils and neutrophil-to-lymphocyte ratio (NLR) values indicates a negative correlation in the normal control group. We found no significant correlation between the normal control group’s FBG or glycated albumin and hematological parameters (Table-3).

**Table-3:** Correlation of FBG, insulin, and glycated albumin versus hematological indices in normal control rats.

Variables	Correlation with FBG		Correlation with Insulin		Correlation with glycated albumin	
		
	r-value	p-value	r-value	p-value	r-value	p-value
White blood cells (10^3^/μL)	−0.01423	0.9710	−0.6105	0.0808	0.07277	0.8524
Lymphocytes (10^3^/μL)	−0.5864	0.1843	0.6473	0.0595	0.1033	0.7913
Neutrophil (10^3^/μL)	0.1350	0.7291	−0.7414*	0.0222	−0.1255	0.7476
Erythrocytes (10^6^/μL)	−0.03061	0.9377	−0.2817	0.4627	0.1210	0.7565
Hemoglobin (g/dL)	−0.04421	0.9101	−0.3571	0.3454	0.04242	0.9137
Hematocrit (%)	−0.05701	0.8842	−0.2681	0.4856	0.1141	0.7701
MCV (fL)	−0.06650	0.8650	0.3913	0.2976	−0.04076	0.9171
MCH (pg)	0.1301	0.7387	−0.00940	0.9808	−0.3227	0.4418
MCHC (g/dL)	−0.00050	0.9990	−0.5409	0.1349	−0.3480	0.3588
RDW (fL)	−0.1364	0.7264	−0.2269	0.5571	0.2413	0.5317
Platelet (10^3^/μL)	0.07835	0.8412	−0.3249	0.3936	−0.09813	0.8017
Neutrophil to lymphocyte ratio	0.2784	0.4682	−0.7869*	0.0119	−0.1152	0.7680

Data are expressed in Mean±SD.

* Statistically significant (p<0.05) versus control group. SD=Standard deviation, FBG=Fasting blood glucose, MCV=Mean corpuscular volume, MCH=Mean corpuscular hemoglobin, MCHC=Mean corpuscular hemoglobin concentration, RDW=Red cell distribution width

In the prediabetic group, the correlation of FBG achieved a significant positive correlation with the lymphocyte values (p=0.0002; r=0.9385) and negative correlation with neutrophil (p=0.0004; r=−0.9228). In comparison, the NLR achieved a significant positive correlation (p=0.0001; r=-0.9538) with FBG among prediabetic rats. Following Pearson’s correlation analysis, the WBC was the only parameter found to be negatively correlated with glycated albumin (p=0.0185; r=-0.7559). No other correlations were found on the hematological parameters and insulin level in the prediabetic group (Table-4).

**Table-4:** Correlation of FBG, insulin, and glycated albumin with hematological parameters in prediabetic rats.

Variables	Correlation with FBG		Correlation with Insulin		Correlation with glycated albumin	
		
	r-value	p-value	r-value	p-value	r-value	p-value
White blood cells (10^3^/μL)	0.1631	0.6750	−0.2509	0.5149	−0.7559*	0.0185
Lymphocytes (10^3^/μL)	0.9385**	0.0002	0.02245	0.9543	0.03215	0.9346
Neutrophil (10^3^/μL)	−0.9228**	0.0004	0.1894	0.6255	0.3074	0.4211
Erythrocytes (10^6^/μL)	0.5446	0.1295	−0.06184	0.8744	−0.4500	0.2242
Hemoglobin (g/dL)	0.5291	0.1430	−0.1295	0.7399	−0.3991	0.2872
Hematocrit (%)	0.5731	0.1067	−0.07789	0.8421	−0.3009	0.4313
MCV (fL)	−0.1980	0.6096	−0.2049	0.5969	0.2277	0.5557
MCH (pg)	−0.02157	0.9561	−0.08857	0.8210	0.3181	0.3988
MCHC (g/dL)	−0.4108	0.4444	0.5500	0.2222	0.2063	0.7778
RDW (fL)	0.05894	0.8803	0.4238	0.2556	0.1403	0.7189
Platelet (10^3^/μL)	0.3985	0.2881	−0.4749	0.1964	−0.3439	0.3649
NLR	−0.9538***	0.0001	0.1142	0.7698	0.2229	0.5644

Data are expressed in Mean±SD.

* p<0.05 versus control

** p<0.005 versus control

*** p<0.0001 versus control. FBG=Fasting blood glucose, MCV=Mean corpuscular volume, MCH=Mean corpuscular hemoglobin, MCHC=Mean corpuscular hemoglobin concentration, RDW=Red cell distribution width, NLR=Netrophil to lymphocyte ratio

## Discussion

The routine hematological count had been proven as a good marker in patients with diabetes due to monitoring and management. The goal of the present study was to determine whether there was a correlation between hematological and glucose control parameters in prediabetic rats.

This study found a significant decrease in the RBC indices, such as RBCs, Hb, Hct, and red RDW, in prediabetic rats compared with those in healthy control rats. In contrast, earlier study reported a significant increment in RBC indices, such as RBCs, Hb, Hct, and RDW, in prediabetic rats compared with healthy control rats. This condition is probably caused by a state of hyperinsulinemia, where insulin acts as a cofactor in the erythropoiesis process, resulting in an increase in the RBC value [13].

Pathophysiological disorders in the development of diabetic microangiopathy may be caused by the rheological conditions of RBCs and reduced deformability of RBCs [14]. In patients with T2DM, RBCs are more accessible to aggregate than in healthy individuals. This syndrome may cause microangiopathy by adding sufficient blood viscosity, which harms the microcirculation of patients with diabetes. Chronic hyperglycemia promotes nonenzymatic glycosylation of membrane proteins in RBCs and triggers disruption of the RBC [15]. Another study also found a tendency of RBC counts to decrease in insulin-resistant subjects. However, no statistical significance was found compared with the healthy control [16]. The RBC, Hb, and Hct levels were not statistically significantly different between the prediabetic and diabetic groups [6]. Decreased Hb values are mainly a common symptom of diabetes in those with decreased renal function or albuminuria [17].

The present study also combines an HFD-G diet with a low dosage of STZ to induce prediabetes. Investigators have long used STZ to produce a diabetic model in rodents. STZ was known to be toxic on pancreatic b-cells and can induce irreversible damage to pancreatic islets through deoxyribonucleic acid (DNA) damage because of free radical generation [18]. STZ administration in rats with doses of 40-70 mg/kg BW may result from hyperglycemia within 72 h. STZ administration in rats can also damage the liver, kidney, and intestine. Although some references state that small doses of STZ have minimal toxic effects on organs, STZ can still cause problems in several organs, including the kidneys. STZ can induce nephropathy in rats within 8 weeks after injection [19]. Several studies have reported that feeding rats with an HFD can cause several systemic disturbances, metabolic syndrome, and kidney injuries [20].

The rodent model of metabolic syndrome and kidney failure in mice has been successfully developed by giving an HFD containing 30% fat for 20 weeks [21]. The HFD-induced metabolic syndrome resulted in reduced Hb levels, albuminuria, and impaired sodium handling. The reduction in the RBC count may be explained by the effects of insulin resistance in prediabetes, where insulin has a role in regulating erythropoiesis [22].

RDW measures the range of RBC volume variation and is reported as part of a standard complete blood count. In this study, the RDW values achieve a statistically significant difference between the prediabetic and normal groups. RDW is a marker of prognosis and inflammation in patients with diabetes [23]. In the present research, a reduced RDW was linked to a significantly higher risk of developing diabetes. Low RDW in this study was also associated with high glucose levels, larger waist circumference, and elevated insulin levels [24].

This study also observed a significant difference in the platelet count between the prediabetic and normal groups. Our finding is similar to the previous studies showing increased platelet counts in the diabetes group versus the control [25-28]. Hyperglycemic conditions can increase superoxide production by the mitochondrial electron transport chain, inhibit the activation of endothelial nitric oxide synthase, activate protein kinase C, and activate nuclear factor-kappa B (NF-kB) [29]. This condition causes an increase in reactive oxygen specimens resulting in DNA damage. An increased NF-kB level induces inflammatory cytokine production, increased leukocyte-attracting chemokines, and expressions of cell adhesion molecules. These alterations in endothelial cells and monocytes may lead to an increase in tissue factor synthesis, the primary procoagulant seen in atherosclerotic plaque, as well as platelet activation and aggregation, and changes in coagulation and fibrinolytic factors [30]. Studies suggested that platelet aggregation, activation, and immaturity are all enhanced in diabetes [7]. Reports had indicated that in prediabetics, higher von Willebrand factor levels were found. The von Willebrand factor is one of the essential factors for platelet aggregation and adhesion [31].

This study determined a correlation of hematological indices with FBG, insulin, and glycated albumin level. In this study, lymphocytes, neutrophils, and the NLR ratio were significantly correlated with the FBG level in the prediabetic group. The NLR was determined by dividing the absolute neutrophil and lymphocyte counts from the same blood test [32]. Chronic inflammation is essential in the development of diabetes and its cardiovascular implications [33]. The NLR is frequently used as a measure in systemic inflammation [34].

In contrast, the value of lymphocytes in this study decreased slightly, although the number was not significant. The minimal changes in number may be related to chronic inflammatory conditions in the prediabetes group, which can increase pro-inflammatory mediators. This finding is similar to the studies in that NLR explained a significant increase in prediabetic and diabetic subjects [35-38]. These inflammatory mediators will bind to receptors on the surface of lymphocytes, thereby initiating apoptosis. This condition causes lymphocytopenia in subjects with prediabetes or diabetes [39,40]. Increased neutrophil levels can cause insulin resistance through the mechanism of increasing inflammation. An elevated NLR is associated with increased pro-inflammatory levels, leading to persistent neutrophil activation [41]. In addition, lymphocytes may be associated with inflammation. Previous study by NIshimura *et al*. [42] have shown that insulin resistance may be associated with T cell-mediated signal transduction resulting in a decrease in T cell count.

In the present study, we found a negative correlation between WBCs and glycated albumin in the prediabetes rats. In contrast with this result, a study by Xu *et al*. [43] showed that glycated albumin levels were positively associated with WBC levels within diabetic conditions.

Evidence indicates that increased WBCs are an independent predictor of diabetes mellitus progression. However, the mechanism underlying this increase remains undetermined [44,45].

## Conclusion

We conclude that hematological parameters are significantly decreased in a prediabetic rat model, such as RBC, Hb, Hct, and RDW. At the same time, there is a strong negative correlation between FBG and NLR. Our finding indicates that in hyperglycemia, there are ongoing inflammatory reactions. In prediabetic situations, hematological markers may provide insight into how well an individual manages their hyperglycemic condition. In the future, hematological indices could be useful indicators of glycemic control in prediabetic conditions. The study’s limitations include the small sample size, duration of prediabetes, and inability to evaluate a cause-effect relationship between variables.

## Authors’ Contributions

DGBK, VS, and ML: Contributed to the conception and design of the study, data collection, and manuscript writing. EHP and TJET: Supervised the data collection and analysis. ML was responsible for funding acquisition. All authors read and approved the final manuscript.
